# Changing Perceptions of Harm of e-Cigarette vs Cigarette Use Among Adults in 2 US National Surveys From 2012 to 2017

**DOI:** 10.1001/jamanetworkopen.2019.1047

**Published:** 2019-03-29

**Authors:** Jidong Huang, Bo Feng, Scott R. Weaver, Terry F. Pechacek, Paul Slovic, Michael P. Eriksen

**Affiliations:** 1 Department of Health Policy and Behavioral Sciences, School of Public Health, Georgia State University, Atlanta; 2 Andrew Young School of Policy Studies, Georgia State University, Atlanta; 3 Currently with IMPAQ International, Columbia, Maryland; 4 Department of Population Health Sciences, School of Public Health, Georgia State University; 5 Decision Research, Eugene, Oregon; 6 Department of Psychology, University of Oregon, Eugene

## Abstract

**Importance:**

Debate is ongoing about whether the scientific evidence of the health risks of electronic cigarettes (e-cigarettes) compared with combustible cigarettes (hereinafter referred to as cigarettes) has been accurately communicated to the public. Large representative surveys are needed to examine how the public perceives the health risk of e-cigarettes and how their perceptions change over time.

**Objective:**

To examine how US adults perceived the harm of e-cigarettes relative to cigarettes and how their perception has changed from 2012 to 2017.

**Design, Setting, and Participants:**

Survey study using data from 2 multiyear cross-sectional nationally representative surveys—the Tobacco Products and Risk Perceptions Surveys (TPRPS) and the Health Information National Trends Surveys (HINTS)—to assess perceived harm of e-cigarettes relative to cigarettes among US adults in 2012, 2014, 2015, 2016, and 2017. Respondents were selected via address-based sampling or random-digit dialing and consisted of adults 18 years or older. Analyses were conducted from February through April 2018.

**Main Outcomes and Measures:**

Self-reported perceived harm of e-cigarettes relative to cigarettes.

**Results:**

The analytical samples of TPRPS consisted of 2800 adults in 2012 (cumulative response rate, 7.3%), 5668 in 2014 (cumulative response rate, 6.6%), 5372 in 2015 (cumulative response rate, 6.8%), 5245 in 2016 (cumulative response rate, 6.4%), and 5357 in 2017 (cumulative response rate, 5.8%). The analytical samples of HINTS consisted of 2609 adults in 2012 (response rate, 39.9%), 3301 in 2014 (response rate, 34.4%), 2224 in 2015 (response rate, 33.0%), and 2683 in 2017 (response rate, 32.4%). The proportion of adults who perceived e-cigarettes as less harmful than cigarettes decreased from 39.4% (95% CI, 36.9%-41.9%) in 2012 to 33.9% (95% CI, 32.7%-35.2%) in 2017 in TPRPS and decreased from 50.7% (95% CI, 48.8%-52.7%) in 2012 to 34.5% (95% CI, 32.7%-36.3%) in 2017 in HINTS. During the same period, the proportion of adults who perceived e-cigarettes to be as harmful as cigarettes increased from 11.5% (95% CI, 10.0%-13.2%) in 2012 to 36.4% (95% CI, 35.1%-37.7%) in 2017 (TPRPS) and from 46.4% (95% CI, 44.5%-48.3%) in 2012 to 55.6% (95% CI, 53.7%-57.5%) in 2017 (HINTS). Those who perceived e-cigarettes to be more harmful than cigarettes increased from 1.3% (95% CI, 0.8%-2.2%) in 2012 to 4.3% (95% CI, 3.8%-4.9%) in 2017 (TPRPS) and from 2.8% (95% CI, 2.2%-3.5%) in 2012 to 9.9% (95% CI, 8.8%-11.1%) in 2017 (HINTS).

**Conclusions and Relevance:**

In this study, the proportion of US adults who perceived e-cigarettes to be as harmful as or more harmful than cigarettes increased substantially from 2012 to 2017. The findings of this study underscore the urgent need to accurately communicate the risks of e-cigarettes to the public, which should clearly differentiate the absolute from the relative harms of e-cigarettes.

## Key Points

**Questions:**

How do US adults perceive the harm of electronic cigarettes (e-cigarettes) relative to combustible cigarettes, and how has their perception changed over time?

**Findings:**

In 2 nationally representative multiyear cross-sectional surveys of US adults, the proportion who perceived e-cigarettes to be as harmful as or more harmful than cigarettes increased substantially from 2012 to 2017.

**Meaning:**

The need for accurate communication of the risk of e-cigarettes to the public is urgent and should clearly differentiate the absolute from the relative harm of e-cigarettes.

## Teaser

This survey study of data from 2 cross-sectional nationally representative surveys examines how US adults perceive the harm of electronic cigarettes (e-cigarettes) relative to combustible cigarettes and how their perception has changed from 2012 to 2017.

## Introduction

Although combustible cigarettes (hereinafter referred to as cigarettes) continue to dominate the tobacco product market in the United States, the emergence and rapid growth of electronic cigarettes (e-cigarettes) in the past decade has led to a transformative change in the US tobacco market—a rapid increase in awareness and use of e-cigarettes among youth and adults that coincided with a decline in cigarette smoking.^1,2,3^ This transformation has been accelerated in recent years by the emergence of new generations of e-cigarettes, such as JUUL e-cigarettes (JUUL Labs).^4,5^ The exponential growth in e-cigarettes has prompted a renewed interest in the tobacco harm reduction approach, which aims to curb the smoking epidemic rapidly by encouraging smokers to switch to low-risk tobacco products such as e-cigarettes.^6^ The potential role that e-cigarettes may play in reducing the harm caused by tobacco is still the subject of heated debate.^6,7^

Although the long-term health effects of e-cigarettes are still unknown, growing evidence and consensus among scientists and researchers suggest that the short-term health risks of completely switching to e-cigarettes are substantially less than those of continued smoking for adults who are unable or unwilling to quit cigarette smoking. For example, a recent comprehensive review concluded that, for adult smokers, a complete switch to e-cigarettes would impose substantially less harm than continuing to smoke cigarettes,^8^ which remain the deadliest tobacco product.^9^ Despite the growing scientific evidence, whether the short-term relative health risks of using e-cigarettes compared with cigarette smoking have been accurately communicated to the public is unclear. Some researchers have voiced criticism that the dominant public health message in the United States still focuses on the absolute risks of using tobacco products rather than the relative health risks between low-risk products and cigarettes.^10^ Although the US Food and Drug Administration (FDA) has acknowledged that nicotine, which is highly addictive, is delivered through products that represent a continuum of risk,^11^ the FDA does not have active campaigns to communicate this message to the public. As such, the risk communication of different tobacco and nicotine products was largely left in the hands of tobacco industry, the e-cigarette industry, and the media.

Previous research has demonstrated that perception of risk plays a critical role in decisions to use tobacco.^12^ For example, studies have shown that concerns about the health risks are one of the most cited reasons to quit smoking among current and former smokers.^13,14^ Similarly, consumers’ risk perception about e-cigarettes and other new and emerging tobacco products may also play an important role in influencing how these products are used and who will use these products. For example, one of the common cited reasons for e-cigarette use is the belief that e-cigarettes are less harmful than cigarettes.^15,16,17^ A recent comprehensive review of consumers’ relative risk perceptions about different tobacco products found that, among e-cigarette users, most respondents perceived e-cigarettes as less harmful than cigarettes.^18^ In addition, studies have also shown that, in certain population groups, the perception of e-cigarettes as less harmful was associated with future use of e-cigarettes.^19^

Although research on the relative risk perception between e-cigarettes and cigarettes is growing, many studies tend to focus on perception at a specific point, in a specific geographic area, and among a specific subpopulation.^18^ Little is known about how the overall risk perception of e-cigarettes has evolved or changed over time. In addition, previous studies^20^ used different approaches to measure risk perception of e-cigarettes, making findings difficult to compare across studies and over time. In the present study, we examine whether and to what extent the perceived relative harm of e-cigarettes compared with cigarettes has changed during a 6-year period (2012-2017) in the United States. This study builds on previous research^21^ and uses data from 2 nationally representative surveys of noninstitutionalized US adults to examine the perceived relative harm of e-cigarettes compared with cigarettes and how the perception has evolved over time.

## Methods

The Tobacco Products and Risk Perceptions Surveys (TPRPS) were conducted by The Tobacco Center of Regulatory Science at the School of Public Health, Georgia State University, Atlanta, in 2012, 2014, 2015, 2016, and 2017. TPRPS asked US adults about their perception of the risk of e-cigarettes relative to cigarettes. The Health Information National Trends Surveys (HINTS) of the National Cancer Institute, Rockville, Maryland, collect nationally representative data about the US public’s knowledge of, attitudes toward, and use of cancer- and health-related information and track changes in the rapidly evolving health communication and information technology landscape. The target population of HINTS consists of adults 18 years or older in the US civilian noninstitutionalized population. For comparison with TPRPS, we used data from HINTS 4, cycle 2 (conducted in 2012); HINTS 4, cycle 4 (conducted in 2014); HINTS-FDA (conducted in 2015); and HINTS 5, cycle 1 (conducted in 2017). HINTS was not conducted in 2016. This study received approval from the institutional review board at Georgia State University and followed the American Association for Public Opinion Research (AAPOR) reporting guidelines. This study did not require participant informed consent because of prior consent to participate in the TPRPS and HINTS surveys.

### Data Sources

#### Tobacco Products and Risk Perceptions Surveys

The TPRPS sample was drawn from KnowledgePanel, a probability-based online research panel designed to be representative of noninstitutionalized US adults (18 years or older). KnowledgePanel’s eligibility is limited to those who were sampled via address-based sampling or random-digit dialing. A computer with internet access is provided to those recruited panelists without internet access. The sample used in this study combined a probability sample of US adults from KnowledgePanel and a representative oversample of preidentified cigarette smokers. A total of 4170 respondents completed TPRPS in 2012 (cumulative response rate, 7.3%), 5717 in 2014 (cumulative response rate, 6.6%), 6051 in 2015 (cumulative response rate, 6.8%), 6014 in 2016 (cumulative response rate, 6.4%), and 5992 in 2017 (cumulative response rate, 5.8%). We constructed a poststratification weight specific to this study using an iterative proportional fitting procedure (raking) to adjust for survey nonresponse as well as for oversampling of smokers. The benchmarks for adjustment were based on demographic and geographic distributions from the most recent Current Population Surveys for the respective survey years. The raking factors included age, sex, race/ethnicity, household income, educational attainment, US Census region, metropolitan area, and internet access. More details on these surveys can be found in previous publications.^22,23^ Key demographic characteristics of the samples are shown in the eTables 1 through 5 in the Supplement.

#### Health Information National Trends Surveys

 The sampling strategy for HINTS consisted of a 2-stage design. In the first stage, a stratified sample of addresses was selected from a file of residential addresses. In the second stage, 1 adult was selected within each sampled household. The sampling frame of addresses was grouped in areas with high and with low concentrations of minority populations. An equal-probability sample of addresses was selected from within each explicit sampling stratum. The Next Birthday Method^38^ was used to select the 1 adult in each household.

HINTS 4, cycle 2 (2012), surveyed 3630 US respondents (response rate, 39.9%); HINTS 4, cycle 4 (2014), 3677 US respondents (response rate, 34.4%); HINTS FDA (2015), 3787 US respondents (response rate, 33.0%); and HINTS 5, cycle 1 (2017), 3285 US respondents (response rate, 32.4%). Although the same individuals may be surveyed multiple times, HINTS was designed to be nationally representative cross-sectional surveys. The final replicate weights, which account for sampling stratum, nonresponse adjustments, and calibration adjustments, were used in our analyses of the HINTS data. HINTS was conducted exclusively by mail. More details on sampling methods of HINTS can be found elsewhere.^24^

### Measures

Perceived harm of e-cigarettes relative to cigarettes was assessed in TPRPS using 2 questions. The first question was “Have you ever heard of e-cigarettes/electronic vapor products?” For those who answered “yes,” a follow-up question was asked: “Is using e-cigarettes/electronic vapor products less harmful, about the same level of harm, or more harmful than smoking regular cigarettes?” Four response categories were constructed based on the answer to the second question: “less harmful,” “about the same level of harm,” “more harmful,” and “I don’t know,” the last of which captures the uncertainty in risk perception.^25^ Respondents who had missing responses for these 2 key measures were dropped from analysis.

Perceived harm of e-cigarettes relative to cigarettes was assessed in HINTS using the following question: “Compared to smoking cigarettes, would you say that electronic cigarettes are… (1) much less harmful; (2) less harmful; (3) just as harmful; (4) more harmful; (5) much more harmful; or (6) I’ve never heard of electronic cigarettes.” Based on the answer to this question, 3 response categories were constructed for those who have heard of e-cigarettes: “less harmful (including options 1 and 2),” “about the same level of harm [option 3],” and “more harmful (including options 4 and 5).” Those who indicated that they have never heard of electronic cigarettes were dropped from analysis. In addition, respondents who had missing responses for this question were dropped from analysis. In 2015 HINTS-FDA, the response category “I don’t know” was added to this question. Therefore, the 2015 HINTS classification has 1 more category than those of other years. To be consistent with other waves of HINTS, we dropped those who responded “I don’t know” in the analysis of 2015 HINTS-FDA data.

### Statistical Analysis

Analyses were conducted from February through April 2018 using Stata software (version 14; StataCorp) to obtain design-based (weighted) point estimates and 95% CIs of the response category proportions for the risk perception items discussed above. We used the Mann-Kendall test for monotonic trend to conduct statistical tests for trend analysis. We used the Wilcoxon-Mann-Whitney test to examine the year-over-year differences. The level of statistical significance used was *P* = .05 for 2-sided tests.

## Results

For TPRPS, the number of adult survey participants in our analytical samples was 2800 in 2012, 5668 in 2014, 5372 in 2015, 5245 in 2016, and 5357 in 2017, respectively. The analytical samples of HINTS consisted of 2609 adults in 2012, 3301 adults in 2014, 2224 adults in 2015, and 2683 adults in 2017.

**Figure 1.**
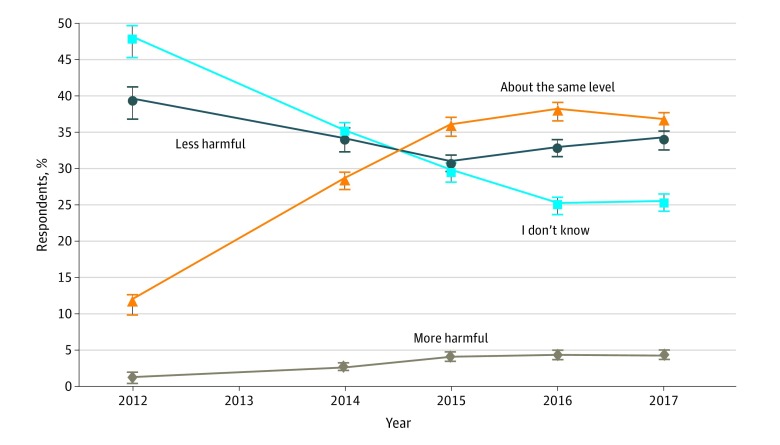
Perceived Harm of Electronic Cigarettes Relative to Combustible Cigarettes in the Tobacco Products and Risk Perceptions Surveys The survey was not conducted in 2013. Percentages are weighted. Error bars indicate 95% CIs.

Figure 1 depicts the perceived harm of e-cigarettes relative to cigarettes using the TPRPS data. The proportion of adults who perceived e-cigarettes as less harmful declined from 39.4% (95% CI, 36.9%-41.9%) in 2012 to 33.9% (95% CI, 32.7%-35.2%) in 2017. This decline occurred primarily from 2012 to 2015. Since 2015, the proportion of adults who perceived e-cigarettes to be less harmful than cigarettes has stabilized at approximately 33%.

During the same period, the proportion of adults who perceived e-cigarettes as equally harmful as cigarettes more than tripled from 11.5% (95% CI, 10.0%-13.2%) in 2012 to 36.4% (95% CI, 35.1%-37.7%) in 2017. This increase occurred primarily from 2012 to 2015. Since 2015, the proportion of adults who perceived e-cigarettes as harmful as cigarettes has hovered at approximately 36%.

Similarly, the proportion of adults who perceived e-cigarettes to be more harmful than cigarettes also more than tripled from 1.3% (95% CI, 0.8%-2.2%) in 2012 to 4.3% (95% CI, 3.8%-4.9%) in 2017. This increase also occurred mainly from 2012 to 2015. Since 2015, the proportion of adults who perceived e-cigarettes to be more harmful than cigarettes has remained at the 4.1% level with no substantial changes.

Over time, more adults have formed some opinions about the risk of e-cigarettes relative to cigarettes, as evidenced by the decline in the “I don’t know” category from 47.8% (95% CI, 45.3%-50.3%) in 2012 to 25.3% (95% CI, 25.3%-26.5%) in 2017. The decline was most pronounced from 2012 to 2015; since then, the drop in this category has decelerated.

**Figure 2.**
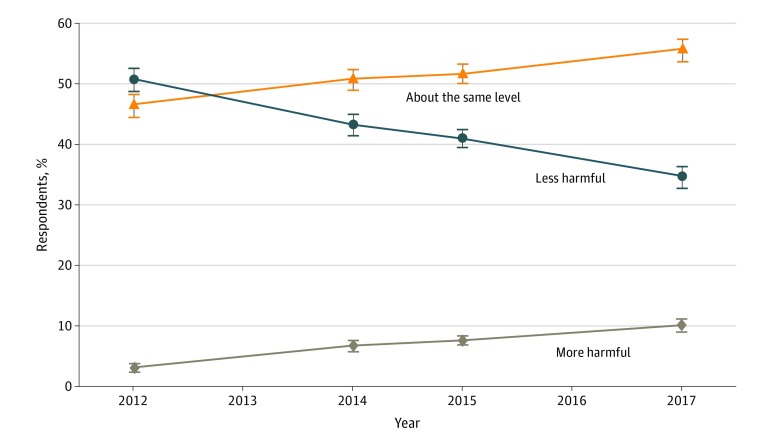
Perceived Harm of Electronic Cigarettes Relative to Combustible Cigarettes in the Health Information National Trends Surveys The survey was not conducted in 2016. Percentages are weighted. Error bars indicate 95% CIs.

Figure 2 depicts the perceived harmfulness of e-cigarettes relative to cigarettes using the HINTS data. Unlike the TPRPS data, the HINTS data do not have the “I don’t know” category, with the exception of the 2015 HINTS responses. Consequently, the estimated proportions of US adults who perceived e-cigarettes as less harmful, equally harmful, or more harmful than cigarettes based on the HINTS data are different from those based on the TPRPS data. However, the changes in risk perception over time are consistent between both data sources.

Overall, in the HINTS data, the proportion of adults who perceived e-cigarettes as less harmful than cigarettes declined from 50.7% (95% CI, 48.8%-52.7%) in 2012 to 34.5% (95% CI, 32.7%-36.3%) in 2017. Similar to Figure 1, this decline occurred primarily from 2012 to 2015. However, the proportion of adults who perceived e-cigarettes to be equally harmful as cigarettes increased from 46.4% (95% CI, 44.5%-48.3%) in 2012 to 55.6% (95% CI, 53.7%-57.5%) in 2017. This increase occurred primarily from 2012 to 2014. The proportion of adults who perceived e-cigarettes as more harmful than cigarettes more than tripled from 2.8% (95% CI, 2.2%-3.5%) in 2012 to 9.9% (95% CI, 8.8%-11.1%) in 2017.

Table 1 presents perceived harm of e-cigarettes relative to cigarettes by tobacco use status based on the TPRPS data. Compared with respondents who never smoked (never smokers), former smokers, and current smokers, the percentage of e-cigarette users and dual users who perceived e-cigarettes were less harmful than cigarettes was substantially and consistently higher across all survey years. Among never, former, and current smokers, the percentage of those who perceived e-cigarettes as equally harmful or more harmful than cigarettes increased significantly from 2012 (never smokers, 13.8% [95% CI, 11.6%-15.3%]; former smokers, 11.9% [95% CI, 9.9%-14.0%]; current smokers, 11.7% [95% CI, 8.3%-15.3%]) to 2017 (never smokers, 41.8% [95% CI, 38.0%-45.2%]; former smokers, 39.6% [95% CI, 36.1%-43.0%]; current smokers, 39.4% [95% CI, 35.0%-43.5%]). The proportion of never and current smokers who perceived e-cigarettes as less harmful than cigarettes decreased significantly from 2012 (never smokers, 39.4% [95% CI, 36.9%-41.9%]; current smokers, 44.7% [95% CI, 40.4%-49.0%]) to 2017 (never smokers, 34.9% [95% CI, 33.1%-36.7%]; current smokers, 33.5% [95% CI, 30.8%-36.2%]). Among former smokers, however, no significant decline occurred in the percentage of those who perceived e-cigarettes as less harmful than cigarettes during the same period (34.9% [95% CI, 31.7%-38.2%] in 2012 vs 32.5% [95% CI, 30.1%-34.8%] in 2017). In contrast to never and current smokers, the proportion of e-cigarette users and dual users who perceived e-cigarettes as less harmful than or equally harmful as cigarettes did not change significantly from 2014 to 2017. Similar to never, current, and former smokers, the percentages of e-cigarette and dual users who perceived e-cigarettes as more harmful than cigarettes also increased significantly from 2014 (e-cigarette users, 1.6% [95% CI, 0.2%-3.0%]; dual users, 2.3% [95% CI, 0.5%-4.2%]) to 2017 (e-cigarette users, 5.3% [95% CI, 3.2%-7.5%]; dual users, 6.1% [95% CI, 3.1%-9.2%]).

**Table 1.** Perceived Harm of e-Cigarettes Relative to Combustible Cigarettes in the Tobacco Products and Risk Perceptions Surveys

Perception of e-Cigarettes vs Cigarettes	Survey Year, % of Respondents (95% CI)^a^				
	2012	2014	2015	2016	2017
**Never Smokers**					
Less harmful	39.4 (36.9-41.9)	33.6 (31.8-35.3)	30.5 (28.7-32.2)	33.4 (31.6-35.2)	34.9 (33.1-36.7)
About the same level	12.1 (10.5-13.8)	31.2 (29.5-32.9)	37.8 (35.9-39.6)	40.6 (38.7-42.5)	37.4 (35.6-39.2)
More harmful	1.7 (1.1-2.4)	2.8 (2.2-3.4)	4.1 (3.4-4.9)	4.5 (3.7-5.3)	4.4 (3.6-5.1)
Do not know	46.8 (44.2-49.3)	32.4 (30.7-34.1)	27.7 (26.0-29.4)	21.5 (19.9-23.1)	23.4 (21.8-25.0)
**Former Smokers**					
Less harmful	34.9 (31.7-38.2)	32.1 (29.8-34.5)	28.3 (26.0-30.6)	30.9 (28.5-33.3)	32.5 (30.1-34.8)
About the same level	10.8 (8.6-12.9)	24.5 (22.3-26.6)	34.1 (31.7-36.5)	34.8 (32.4-37.2)	35.9 (33.5-38.3)
More harmful	1.1 (0.4-1.8)	2.6 (1.8-3.3)	4.0 (3.0-5.0)	4.0 (3.0-5.0)	3.7 (2.7-4.6)
Do not know	53.2 (49.8-56.6)	40.9 (38.4-43.3)	33.5 (31.2-35.9)	30.3 (28.0-32.7)	27.9 (25.7-30.2)
**Current Smokers**					
Less harmful	44.7 (40.4-49.0)	37.7 (35.1-40.3)	36.0 (33.3-38.8)	34.0 (31.2-36.7)	33.5 (30.8-36.2)
About the same level	11.0 (8.3-13.8)	24.5 (22.2-26.8)	30.8 (28.2-33.4)	33.2 (30.5-35.9)	33.9 (31.2-36.6)
More harmful	0.7 (0.0-1.4)	2.9 (2.0-3.8)	4.3 (3.1-5.4)	5.0 (3.7-6.2)	5.5 (4.2-6.9)
Do not know	43.5 (39.2-47.9)	34.9 (32.3-37.4)	28.9 (26.3-31.5)	27.9 (25.4-30.5)	27.1 (24.5-29.6)
**Current e-Cigarette Users**					
Less harmful	NA	60.1 (54.7-65.4)	63.2 (58.5-68.0)	66.6 (61.1-72.1)	61.6 (56.9-66.3)
About the same level	NA	20.4 (16.0-24.8)	14.8 (11.3-18.3)	22.4 (17.6-27.3)	20.0 (16.1-23.8)
More harmful	NA	1.6 (0.2-3.0)	5.3 (3.1-7.5)	2.0 (0.4-3.7)	5.3 (3.2-7.5)
Do not know	NA	18.0 (13.8-22.1)	16.6 (13.0-20.3)	9.0 (5.6-12.3)	13.1 (9.8-16.4)
**Dual Users (Cigarettes and e-Cigarettes)**					
Less harmful	NA	56.5 (50.4-62.6)	56.7 (51.0-62.5)	64.2 (57.4-71.0)	51.2 (44.9-57.5)
About the same level	NA	23.4 (18.2-28.6)	18.5 (14.0-23.1)	24.3 (18.2-30.4)	30.1 (24.3-35.9)
More harmful	NA	2.3 (0.5-4.2)	4.5 (2.1-6.9)	3.3 (0.8-5.9)	6.1 (3.1-9.2)
Do not know	NA	17.8 (13.1-22.4)	20.2 (15.5-24.9)	8.2 (4.3-12.1)	12.5 (8.4-16.7)

Abbreviations: e-cigarette, electronic cigarette; NA, not available.

^a^ All percentages are weighted. The 2012 Tobacco Products and Risk Perceptions Surveys did not ask questions about current use of e-cigarettes.

Table 2 presents the perceived harm of e-cigarettes relative to cigarettes by smoking status based on the HINTS data, which did not ask questions about current use of e-cigarettes. Overall, the percentage of never and former smokers who perceived e-cigarettes to be as harmful as cigarettes was consistently and substantially higher than among current smokers across all survey years. In contrast, the percentage of current smokers who perceived e-cigarettes as less harmful than cigarettes was consistently and substantially higher than that among never smokers across all survey years. Regardless of smoking status, the proportion of adults who perceived e-cigarettes as less harmful than cigarettes decreased significantly from 2012 (never smokers, 45.9% [95% CI, 43.3%-48.5%]; former smokers, 49.5% [95% CI, 45.7%-53.2%]; current smokers, 65.0% [95% CI, 60.8%-69.2%]) to 2017 (never smokers, 33.1% [95% CI, 30.8%-35.4%]; former smokers, 33.3% [95% CI, 29.7%-36.6%]; current smokers, 41.9% [95% CI, 36.7%-47.1%]). During the same period, the percentage of adults who perceived e-cigarettes as more harmful than cigarettes increased significantly among all 3 subgroups (2012 among never smokers, 3.5% [95% CI, 2.5%-4.4%]; 2012 among former smokers, 2.4% [95% CI, 1.2%-3.5%]; 2012 among current smokers, 1.8% [95% CI, 0.6%-3.0%]; 2017 among never smokers, 9.1% [95% CI, 7.6%-10.5%]; 2017 among former smokers, 9.3% [95% CI, 7.2%-11.4%]; 2017 among current smokers, 14.2% [95% CI, 10.5%-17.8%]).

**Table 2.** Perceived Harm of e-Cigarettes Relative to Combustible Cigarettes in the Health Information National Trends Surveys

Perception of e-Cigarettes vs Cigarettes	Survey Year, % of Respondents (95% CI)^a^			
	2012	2014	2015	2017
**Never Smokers**				
Less harmful	45.9 (43.3-48.5)	38.3 (36.2-40.5)	36.3 (33.5-39.0)	33.1 (30.8-35.4)
About the same level	50.7 (48.0-53.3)	54.9 (52.7-57.1)	57.9 (55.1-60.7)	57.8 (55.4-60.3)
More harmful	3.5 (2.5-4.4)	6.7 (5.6-7.9)	5.8 (4.5-7.2)	9.1 (7.6-10.5)
**Former Smokers**				
Less harmful	49.5 (45.7-53.2)	45.4 (42.1-48.6)	44.8 (40.9-48.6)	33.3 (29.9-36.6)
About the same level	48.2 (44.4-52.0)	50.7 (47.4-54.0)	45.6 (41.8-49.4)	57.4 (53.8-61.0)
More harmful	2.4 (1.2-3.5)	3.9 (2.6-5.2)	9.7 (7.4-11.9)	9.3 (7.2-11.4)
**Current Smokers**				
Less harmful	65.0 (60.8-69.2)	57.6 (53.1-62.1)	52.0 (46.6-57.4)	41.9 (36.7-47.1)
About the same level	33.2 (29.0-37.4)	33.8 (29.5-38.1)	38.2 (33.0-43.4)	43.9 (38.7-49.2)
More harmful	1.8 (0.6-3.0)	8.6 (6.0-11.1)	9.8 (6.6-13.0)	14.2 (10.5-17.8)

Abbreviation: e-cigarette, electronic cigarette.

^a^ All percentages are weighted. The Health Information National Trends Surveys did not ask questions about current use of e-cigarettes.

## Discussion

Three principal findings emerged from this study. First, both national surveys show that from 2012 to 2017, the proportion of US adults who perceived e-cigarettes as less harmful than cigarettes decreased significantly. Second, during the same period, the perception of e-cigarettes to be equally or more harmful than cigarettes increased significantly. The changes in perception of harm of e-cigarettes relative to cigarettes occurred primarily from 2012 to 2015. From 2015 to 2017, the proportion of US adults who perceived e-cigarettes as equally or more harmful than cigarettes did not change substantially. Third, compared with never, former, and current smokers, a higher proportion of e-cigarette users perceived e-cigarettes as less harmful than cigarettes. However, even among e-cigarette users, the percentage of those who perceived e-cigarette as more harmful than cigarettes increased significantly from 2012 to 2017.

In addition, the results based on the TPRPS data also reveal that, over time, adults who were uncertain about the health risks associated with e-cigarette use were more likely to consider e-cigarettes to be as harmful as or more harmful than cigarettes. A quarter of respondents in 2017 TPRPS (25.3%; 95 CI, 24.1%-26.5%) reported that they did not know the relative risks between e-cigarettes and cigarettes, indicating that a large proportion of the population was still uncertain about the risks of e-cigarette use. This finding suggests that future research or surveys should include questions that capture the uncertainty associated with the risks of e-cigarette use.

Our finding that most US adults were not certain about the health risk of e-cigarettes or perceived e-cigarettes to be as harmful as or more harmful than cigarettes is consistent with the findings from a number of previous studies.^19,21,26,27^ However, this finding differs from that of a recent review,^18^ which concluded that most study respondents perceived that e-cigarettes are less harmful than cigarettes. The studies included in this review analyzed data before 2015, which may explain the discrepancies. As our study shows, before 2015, a large proportion of adults indeed perceived e-cigarettes as less harmful. However, over time, the proportion of US adults who perceived e-cigarettes as less harmful declined significantly. In addition, those who were initially uncertain about the health risk of e-cigarettes tended to form the perception that e-cigarettes were as harmful as or more harmful than cigarettes.

Previous studies have shown that lower levels of perceived harm of e-cigarettes relative to cigarettes were associated with ever trying and current use of e-cigarettes among adults^28,29^ and exclusive e-cigarette use among smokers who completely switched from cigarettes.^30^ Perception of e-cigarette harm may deter current smokers from initiating or continuing use of e-cigarettes. This perception may also deter a complete switch from cigarettes to e-cigarettes among smokers. In light of this possibility, the observed upward trend of perceiving e-cigarettes to be as harmful as or more harmful than cigarettes among US adults warrants heightened attention.

The increase in the proportion of adults who perceived e-cigarettes to be equally or more harmful than cigarettes from 2012 to 2017 may reflect consumers’ concerns about the risk of addiction and/or the uncertainty about e-cigarette’s long-term health effects. Importantly, this increase may reflect the emergence of new evidence of substantial risk of heart^31^ and lung^32^ diseases associated with e-cigarette use, as well as high levels of pulmonary toxic effects^33^ in e-cigarettes. This increase may also be due to frequent media reports that link e-cigarettes and e-liquid to exposure to toxicants,^34^ serious injuries,^35^ and other health-related problems.^36^ In addition, confusion between relative risk and absolute risk of e-cigarettes may contribute to framing bias in risk communication and result in media reports and press releases in which absolute harm is overstated and relative harm is downplayed. Lack of accurate, consistent, and proactive risk communications to the public from scientists may also contribute to the confusion about the health risks of e-cigarettes.

Our findings underscore the urgency to convey accurate risk information about e-cigarettes to the public, especially to adult smokers who are unable or unwilling to quit smoking, and therefore could benefit most by switching from cigarettes to e-cigarettes. Public health messages may be beneficial by appropriately balancing the emphasis on the potential reduction in harm in completely switching to e-cigarette use compared with continued smoking and an accurate interpretation of the absolute harm of e-cigarette use.

### Limitations

Our study has several limitations. In particular, the rapid product innovation and development in the e-cigarette marketplace made it difficult to identify the accurate terminology to develop questions about e-cigarettes. For example, in the 2012 and 2014 TPRPS, a generic term *e-cigarettes* was used to describe the product; however, in 2015, 2016, and 2017 TPRPS, *electronic vapor products* was used to capture newer generations of vaping products and changing consumer terminology. This variation in wording may influence the comparability across years. In addition, the perceived relative harm of e-cigarettes was assessed using a generic question, similar to those of previous studies.^19,21,26,37^ This generic question may not capture various specific aspects of harm or health risks associated with e-cigarette use. The risk perceptions of e-cigarettes associated with specific diseases, such as lung and heart diseases and cancers, may differ from the overall risk perception associated with e-cigarette use.

## Conclusions

We analyzed 2 large nationally representative surveys of US adults to assess the perceived relative harm of e-cigarettes compared with cigarettes and how the perceived relative harm of e-cigarettes evolved from 2012 to 2017. Our analysis revealed a consistent pattern and a change in perceived relative harm of e-cigarettes among US adults in both surveys, which showed that a large proportion of US adults perceived e-cigarettes as equally or more harmful than cigarettes, and this proportion has increased substantially from 2012 to 2017. Given the demonstration by previous studies that perception of risk plays a critical role in decisions to use tobacco, our results imply that at least some smokers may have been deterred from using or switching to e-cigarettes due to the growing perception that e-cigarettes are equally harmful or more harmful than cigarettes. Our results underscore the urgent need for accurate communication of the scientific evidence on the health risks of e-cigarettes and the importance of clearly differentiating the absolute harm from the relative harm of e-cigarettes.

## Notes

Supplement. **eTable 1.** Descriptive Statistics for the Tobacco Products and Risk Perceptions Surveys (TPRPS) and the Health Information National Trends Surveys (HINTS), 2012 **eTable 2.** Descriptive Statistics for the Tobacco Products and Risk Perceptions Surveys (TPRPS) and the Health Information National Trends Surveys (HINTS), 2014 **eTable 3.** Descriptive Statistics for the Tobacco Products and Risk Perceptions Surveys (TPRPS) and the Health Information National Trends Surveys (HINTS), 2015 **eTable 4.** Descriptive Statistics for the Tobacco Products and Risk Perceptions Surveys (TPRPS), 2016 **eTable 5.** Descriptive Statistics for the Tobacco Products and Risk Perceptions Surveys (TPRPS) and the Health Information National Trends Surveys (HINTS), 2017
