# Supplementary material for: Changing Perceptions of Harm of e-Cigarette vs Cigarette Use Among Adults in 2 US National Surveys From 2012 to 2017
Source: JAMA Netw Open. 2019 Mar 29;2(3):e191047. doi: 10.1001/jamanetworkopen.2019.1047 (PMC6450305; doi:10.1001/jamanetworkopen.2019.1047)
Supplement: Supplement. — eTable 1. Descriptive Statistics for the Tobacco Products and Risk Perceptions Surveys (TPRPS) and the Health Information National Trends Surveys (HINTS), 2012 eTable 2. Descriptive Statistics for the Tobacco Products and Risk Perceptions Surveys (TPRPS) and the Health Information National Trends Surveys (HINTS), 2014 eTable 3. Descriptive Statistics for the Tobacco Products and Risk Perceptions Surveys (TPRPS) and the Health Information National Trends Surveys (HINTS), 2015 eTable 4. Descriptive Statistics for the Tobacco Products and Risk Perceptions Surveys (TPRPS), 2016 eTable 5. Descriptive Statistics for the Tobacco Products and Risk Perceptions Surveys (TPRPS) and the Health Information National Trends Surveys (HINTS), 2017 [file jamanetwopen-2-e191047-s001.pdf]

Supplementary Online Content

Huang J, Feng B, Weaver SR, Pechacek TF, Slovic P, Eriksen MP. Changing perceptions of harm of e-cigarette vs cigarette use among adults in 2 US national surveys from 2012 to 2017. *JAMA Netw Open*. 2019;2(3):e191047. doi:10.1001/jamanetworkopen.2019.1047

eTable 1. Descriptive Statistics for the Tobacco Products and Risk Perceptions Surveys (TPRPS) and the Health Information National Trends Surveys (HINTS), 2012

eTable 2. Descriptive Statistics for the Tobacco Products and Risk Perceptions Surveys (TPRPS) and the Health Information National Trends Surveys (HINTS), 2014

eTable 3. Descriptive Statistics for the Tobacco Products and Risk Perceptions Surveys (TPRPS) and the Health Information National Trends Surveys (HINTS), 2015

eTable 4. Descriptive Statistics for the Tobacco Products and Risk Perceptions Surveys (TPRPS), 2016

eTable 5. Descriptive Statistics for the Tobacco Products and Risk Perceptions Surveys (TPRPS) and the Health Information National Trends Surveys (HINTS), 2017

This supplementary material has been provided by the authors to give readers additional information about their work.

© 2019 Huang J et al. *JAMA Network Open.*

eTable 1. Descriptive Statistics for the Tobacco Products and Risk Perceptions Surveys (TPRPS) and the Health Information National Trends Surveys (HINTS), 2012

		TPRPS				HINTS		
		2012						
		Unweighted Frequency, No. (%)	Weighted %	95% CI		Unweighted Frequency, No. (%)	Weighted %	95% CI
E-cigarette use*								
	Current user							
Cigarette use								
	Current user	604 (34.9)	16.7	[15.6,17.9]		586 (16.4)	18.7	[17.4,20.0]
Perceived harmfulness (E-cigs is .. than conventional cigarettes)								
	Less harmful	1,017(36.3)	39.4	[36.9,41.9]		1,222 (46.8)	50.7	[48.8,52.7]
	About the same	350(12.5)	11.5	[10.0,13.2]		1,305 (50.0)	46.4	[44.5,48.3]
	More harmful	29(1.04)	1.3	[0.8,2.2]		82 (3.1)	2.8	[2.2,3.5]
	I don't know[†]	1404(50.14)	47.8	[45.3,50.3]				
Sex								
	Male	1,932 (46.3)	48.2	[46.6,49.7]		1,390 (39.0)	48.6	[46.9,50.2]
	Female	2,238 (53.7)	51.8	[50.3,53.3]		2,171 (61.0)	51.4	[49.8,53.1]
Age								
	18-24	366 (8.8)	12.5	[11.5,13.5]		105 (3.0)	12.9	[11.8,14.0]
	25-34	488 (11.7)	17.5	[16.3,18.6]		424 (12.1)	17.6	[16.4,18.9]
	35-44	740 (17.7)	16.8	[15.6,17.9]		526 (15.0)	17.3	[16.0,18.5]
	45-54	1,000 (24.0)	18.9	17.7,20.1]		720 (20.5)	18.7	[17.4,20.0]
	55-64	750 (18.0)	16.4	[15.3,17.5]		767 (21.9)	16.1	[14.9,17.3]

© 2019 Huang J et al. *JAMA Network Open.*

	65 +	826 (19.8)	17.9	[12.3,19.0]		969 (27.6)	17.4	[16.1,18.6]
Race/Ethnicity								
	White, Non-Hispanic	3,094 (74.2)	67.2	[65.7,68.4]		2,043 (62.7)	67.1	[65.5,68.7]
	Black, Non-Hispanic	406 (9.7)	11.3	[10.3,12.3]		496 (15.2)	10.9	[9.8,12.0]
	Hispanic	429 (10.3)	14.3	13.2,15.3]		511 (15.7)	15.0	[13.7,16.2]
	Other	241 (5.8)	7.2	[6.4,7.9]		208 (6.4)	7.1	[6.2,8.0]
Education								
	< High School	263 (6.3)	12.6	[11.6,13.6]		329 (9.3)	13.5	[12.4,14.6]
	High school graduate	1,074 (25.8)	30.1	[28.7,31.5]		775 (21.9)	20.3	[19.0,21.6]
	Some college	1,334 (32.0)	28.8	[27.4,30.1]		1,057 (29.9)	37.6	[36.0,39.2]
	College +	1,499 (35.9)	28.5	[27.1,29.8]		1,380 (39.0)	28.6	[27.1,30.1]
Household income								
	< 20K	489(11.7)	13.9	[12.9,15.0]		740 (23.5)	21.9	[20.4,23.3]
	20 - 35K	557(13.4)	14.8	[13.7,15.8]		501 (15.9)	14.9	[13.7,16.2]
	35 - 50K	575(13.8)	12.6	[11.6,13.6]		459 (14.6)	15.5	[14.2,16.7]
	50 - 75K	844(20.2)	20.1	[18.8,21.2]		524 (16.6)	16.8	[15.5,18.1]
	75K +	1705(40.8)	38.5	[37.1,40.0]		926 (29.4)	31.0	[29.4,32.6]
Marital status								
	Married	2,482 (58.5)	53.8	[52.3, 55.3]		1,816 (51.5)	53.4	[51.7,55.0]
	Not married	1,688 (40.5)	46.1	[44.6,47.7]		1,712 (48.5)	46.6	[45.0,48.3]
Metro status								
	Non-metro	633 (15.2)	16.1	[15.0,17.2]		543 (15.0)	16.3	[15.1,17.5]

© 2019 Huang J et al. *JAMA Network Open.*

	Metro	3,537 (84.8)	83.9	[82.7,85.0]		3,087 (85.0)	83.7	[82.5,84.9]
*: E-cigarette use was not asked in TPRPS and HINTS in 2012.								
†: This option was asked only in the HINTS-FDA 2015 data. Other HINTS survey did not have this option.								

© 2019 Huang J et al. *JAMA Network Open.*

eTable 2. Descriptive Statistics for the Tobacco Products and Risk Perceptions Surveys (TPRPS) and the Health Information National Trends Surveys (HINTS), 2014

		TPRPS				HINTS			
		2014							
		Unweighted Frequency, No. (%)	Weighted %	95% CI		Unweighted Frequency, No. (%)	Weighted %	95% CI	
E-cigarette use*									
	Current user	331 (6.5)	5.1	[4.5,5.7]					
Cigarette use									
	Current user	1,349 (23.6)	16.6	[15.6,17.5]		498 (13.7)	15.2	[14.0,16.3]	
Perceived harmfulness (E-cigs is .. than conventional cigarettes)									
	Less harmful	1,979 (34.9)	33.8	[32.6,35.1]		1,269 (38.4)	43.1	[41.4,44.8]	
	About the same	1,534 (27.1)	28.3	[27.1,29.4]		1,814 (55.0)	50.6	[48.9,52.3]	
	More harmful	147 (2.6)	2.8	[2.3,3.2]		218 (6.6)	6.4	[5.5,7.2]	
	I don't know[†]	2,008 (35.4)	35.1	[33.9,36.4]					
Sex									
	Male	2,807 (49.1)	48.1	[46.8,49.4]		1,424 (39.5)	48.3	[46.6,49.9]	
	Female	2,910 (50.9)	51.9	[50.6,53.2]		2,184 (60.5)	51.7	[50.1,53.4]	
Age									
	18-24	476 (8.3)	12.6	[11.7,13.5]		102 (2.9)	9.7	[8.7,10.7]	
	25-34	816 (14.3)	16.3	[15.3,17.3]		365 (10.3)	21.1	[19.7,22.4]	
	35-44	940 (16.4)	18.1	[17.1,19.0]		479 (13.7)	17.2	[15.9,18.4]	
	45-54	1,054 (18.4)	16.4	[15.5,17.4]		656 (18.8)	18.9	[17.6,20.2]	
	55-64	1,250 (21.9)	18.9	[17.9,19.9]		828 (23.7)	15.8	[14.6,17.0]	

© 2019 Huang J et al. *JAMA Network Open.*

	65 +	1,181 (20.7)	17.7	[16.8,18.7]		1,065 (30.5)	17.5	[16.2,18.7]	
Race/Ethnicity									
	White, Non-Hispanic	4,221 (73.8)	66.0	[64.8,67.2]		1,960 (59.9)	66.6	[65.0,68.2]	
	Black, Non-Hispanic	566 (9.9)	11.6	[10.7,12.4]		534 (16.3)	11.3	[10.2,12.4]	
	Hispanic	508 (8.9)	15.0	[14.0,15.9]		540 (16.5)	15.1	[13.9,16.4]	
	Other	422 (7.4)	7.5	[6.8,8.2]		239 (7.3)	6.9	[6.0,7.8]	
Education									
	< High School	442 (7.7)	12.6	[11.7,13.4]		308 (8.7)	11.6	[10.6,12.7]	
	High school graduate	1,690 (29.6)	29.6	[28.4,30.8]		670 (19.0)	18.2	[16.9,19.5]	
	Some college	1,735 (30.4)	28.9	[27.8,30.1]		1,090 (30.9)	30.0	[28.5,31.5]	
	College +	1,850 (32.4)	28.9	[27.7,30.1]		1,458 (41.4)	40.2	[38.5,41.8]	
Household income									
	< 20K	814 (14.2)	14.7	[13.7,15.6]		774 (23.6)	19.4	[18.0,20.7]	
	20 - 35K	799 (14.0)	14.0	[13.1,14.9]		489 (14.9)	12.7	[11.6,13.9]	
	35 - 50K	748 (13.1)	12.5	[11.7,13.4]		482 (14.7)	14.8	[13.6,16.0]	
	50 - 75K	1,095 (19.2)	18.4	[17.4,19.4]		550 (16.8)	17.3	[16.0,18.5]	
	75K +	2,261 (39.6)	40.5	[39.2,41.7]		979 (29.9)	35.9	[34.2,37.5]	
Marital status									
	Married	3,067 (53.7)	51.1	[49.8,52.4]		1,807 (51.5)	53.7	[52.1,55.4]	
	Not married	2,650 (46.4)	48.9	[47.6,50.2]		1,699 (48.5)	46.3	[44.6,47.9]	
Metro status									
	Non-metro	899 (15.7)	15.8	[14.9,16.8]		520 (14.1)	16.4	[15.2,17.6]	

© 2019 Huang J et al. *JAMA Network Open.*

	Metro	4,818 (84.3)	84.2	[83.2,85.1]		3,157 (85.9)	83.6	[82.4,84.8]	

*: E-cigarette use was not asked in HINTS in 2014.

†: This option was asked only in the HINTS-FDA 2015 data. Other HINTS survey did not have this option.

© 2019 Huang J et al. *JAMA Network Open.*

eTable 3. Descriptive Statistics for the Tobacco Products and Risk Perceptions Surveys (TPRPS) and the Health Information National Trends Surveys (HINTS), 2015

		TPRPS				HINTS-FDA		
		2015						
		Unweighted Frequency, No. (%)	Weighted %	95% CI		Unweighted Frequency, No. (%)	Weighted %	95% CI
E-cigarette use*								
	Current user	404 (6.7)	5.5	[4.9,6.1]				
Cigarette use								
	Current user	1,284 (21.2)	14.1	[13.2,15.0]		495 (13.5)	14.8	[13.7,16.0]
Perceived harmfulness (E-cigs is .. than conventional cigarettes)								
	Less harmful	1,725 (32.1)	30.7	[29.4,31.9]		873 (24.4)	26.6	[25.2,28.1]
	About the same	1,854 (34.5)	35.7	[34.4,37.0]		1,199 (33.6)	33.7	[32.1,35.2]
	More harmful	183 (3.4)	4.1	[3.6,4.6]		152 (4.3)	4.9	[4.1,5.6]
	I don't know†	1,610 (30.0)	29.5	[28.3,30.8]		1,350 (37.8)	34.8	[33.3,36.4]
Sex								
	Male	3,039 (50.2)	48.6	[47.3,49.8]		1,497 (42.6)	49.1	[47.4,50.7]
	Female	3,012 (49.8)	51.4	[50.2,52.7]		2,018 (57.4)	50.9	[49.3,52.6]
Age								
	18-24	356 (5.9)	9.3	[8.5,10.0]		108 (3.0)	10.6	[9.6,11.6]
	25-34	1,116 (18.4)	20.8	[19.7,21.8]		347 (9.6)	19.8	[18.5,21.1]
	35-44	917 (15.2)	16.2	[15.2,17.1]		428 (11.8)	16.6	[15.3,17.8]
	45-54	958 (15.8)	15.7	[14.8,16.6]		610 (16.8)	17.8	[16.5,19.0]
	55-64	1,262 (20.7)	19.5	[18.5,20.5]		847 (23.4)	16.3	[15.1,17.5]

© 2019 Huang J et al. *JAMA Network Open.*

	65 +	1,442 (23.8)	18.6	[17.6,19.6]		1,288 (35.5)	19.0	[17.7,20.3]
Race/Ethnicity								
	White, Non-Hispanic	4,462 (73.7)	66.1	[64.9,67.3]		2,633 (78.2)	64.9	[63.2,66.5]
	Black, Non-Hispanic	519 (8.6)	11.2	[10.4,12.0]		232 (6.9)	11.4	[10.3,12.4]
	Hispanic	713 (11.8)	15.2	[14.3,16.1]		241 (7.2)	16.1	[14.9,17.4]
	Other	357 (5.9)	7.4	[6.7,8.1]		260 (7.7)	7.7	[6.8,8.6]
Education								
	< High School	372 (6.2)	11.3	[10.5,12.0]		237 (6.5)	10.9	[9.9,11.9]
	High school graduate	2,042 (33.8)	30.1	[28.9,31.2]		727 (19.8)	21.0	[19.7,22.3]
	Some college	1,591 (26.3)	28.9	[27.8,30.0]		1,132 (30.8)	32.8	[31.3,34.4]
	College +	2,046 (33.8)	29.8	[28.6,30.9]		1,578 (43.0)	35.3	[33.7,36.8]
Household income								
	< 20K	918 (15.2)	14.1	[13.2,15.0]		664 (20.1)	20.3	[19.0,21.7]
	20 - 35K	922 (15.2)	13.5	[12.6,14.3]		506 (15.3)	14.8	[13.6,16.0]
	35 - 50K	898 (14.8)	12.8	[12.0,13.6]		415 (12.6)	13.6	[12.4,14.7]
	50 - 75K	1,182 (19.5)	18.3	[17.4,19.3]		605 (18.3)	16.0	[14.8,17.3]
	75K +	2,131 (35.2)	41.3	[40.0,42.5]		1,112 (33.7)	25.3	[33.6,36.9]
Marital status								
	Married	3,368 (55.7)	53.0	[51.7,54.3]		1,932 (52.9)	52.4	[50.8,54.0]
	Not married	2,683 (44.3)	47.0	[45.7,48.3]		1,722 (47.1)	47.6	[46.0,49.2]
Metro status								
	Non-metro	851 (14.1)	15.6	[14.7,16.5]		970 (26.0)	16.6	[15.4,17.8]

© 2019 Huang J et al. *JAMA Network Open.*

	Metro	5,200 (86.0)	84.4	[83.5,85.3]		2,768 (74.1)	83.4	[82.2,84.6]
*: E-cigarette use was not asked in HINTS in 2015.								
†: This option was asked only in the HINTS-FDA 2015 data. Other HINTS survey did not have this option.								

© 2019 Huang J et al. *JAMA Network Open.*

eTable 4. Descriptive Statistics for the Tobacco Products and Risk Perceptions Surveys (TPRPS), 2016

		TPRPS		
		2016		
		Unweighted Frequency, No. (%)	Weighted %	95% CI
E-cigarette use				
	Current user	288 (4.8)	3.8	[3.4,4.3]
Cigarette use				
	Current user	1,288 (21.4)	13.2	[12.3,14.0]
Perceived harmfulness (E-cigs is .. than conventional cigs)				
	Less harmful	1,748 (33.3)	32.8	[31.5,34.0]
	About the same	1,945 (37.1)	37.9	[36.6,39.2]
	More harmful	210 (4.0)	4.4	[3.9,5.0]
	I don't know	1,342 (25.6)	24.9	[23.7,26.1]
Sex				
	Male	3,013 (50.1)	48.0	[46.8,49.3]
	Female	3,001 (49.9)	52.0	[50.7,53.2]
Age				
	18-24	332 (5.5)	8.7	[8.0,9.4]
	25-34	1,044 (17.4)	19.7	[18.7,20.7]
	35-44	818 (13.6)	17.3	[16.3,18.2]
	45-54	978 (16.3)	15.8	[14.9,16.7]
	55-64	1,303 (21.7)	18.5	[17.5,19.5]
	65 +	1,539 (25.6)	20.0	[19.0,21.0]
Race/Ethnicity				
	White, Non-Hispanic	4,434 (73.7)	65.1	[63.8,66.3]
	Black, Non-Hispanic	547 (9.1)	11.8	[11.0,12.6]
	Hispanic	672 (11.2)	15.3	[14.4,16.2]
	Other	361 (6.0)	7.8	[7.1,8.5]
Education				
	< High School	297 (4.9)	10.8	[10.0,11.6]
	High school graduate	1,781 (29.6)	29.3	[28.1,30.4]
	Some college	1,876 (31.2)	28.8	[27.7,30.0]
	College +	2,060 (34.3)	31.1	[30.0,32.3]
Household income				
	< 20K	878 (14.6)	12.7	[11.8,13.5]
	20 - 35K	944 (15.7)	12.9	[12.0,13.7]

© 2019 Huang J et al. *JAMA Network Open.*

	35 - 50K	815 (13.6)	11.4	[10.6,12.2]
	50 - 75K	1,133 (18.8)	17.5	[16.5,18.5]
	75K +	2,244 (37.3)	45.6	[44.4,46.9]
Marital status				
	Married	3,272 (54.4)	55.2	[53.9,56.5]
	Not married	2,742 (45.6)	44.8	[43.5,46.1]
Metro status				
	Non-metro	834 (13.9)	14.1	[13.2,14.9]
	Metro	5,180 (86.1)	85.9	[85.1,86.8]
The Health Information National Trends Surveys (HINTS) was not conducted in 2016.				

© 2019 Huang J et al. *JAMA Network Open.*

eTable 5. Descriptive Statistics for the Tobacco Products and Risk Perceptions Surveys (TPRPS) and the Health Information National Trends Surveys (HINTS), 2017

		TPRPS				HINTS		
		2017						
		Unweighted Frequency, No. (%)	Weighted %	95% CI		Unweighted Frequency, No. (%)	Weighted %	95% CI
E-cigarette use*								
	Current user	416 (6.9)	6.9	[6.3,7.6]				
Cigarette use								
	Current user	1,271 (21.2)	15.0	[14.0,15.9]		414 (12.7)	14.9	[13.7,16.1]
Perceived harmfulness (E-cigs is .. than conventional cigarettes)								
	Less harmful	1,832 (34.2)	33.9	[32.7,35.2]		785 (29.3)	34.5	[32.7,36.3]
	About the same	1,928 (36.0)	36.4	[35.1,37.7]		1,615 (60.2)	55.6	[53.7,57.5]
	More harmful	231 (4.3)	4.3	[3.8,4.9]		283 (10.6)	9.9	[8.8,11.1]
	I don't know†	1,366 (25.5)	25.3	[24.1,26.5]				
Sex								
	Male	2,987 (49.9)	48.1	[46.9,49.4]		1,303 (40.5)	48.9	[47.2,50.6]
	Female	3,005 (50.2)	51.9	[50.6,53.1]		1,914 (59.5)	51.1	[49.4,52.8]
Age								
	18-24	358 (6.0)	8.0	[7.3,8.7]		73 (2.3)	8.3	[7.3,9.2]
	25-34	1,104 (18.4)	20.2	[19.2,21.2]		294 (9.4)	13.6	[12.4,14.8]
	35-44	813 (13.6)	17.5	[16.5,18.5]		407 (12.9)	17.5	[16.2,18.8]
	45-54	933 (15.6)	14.7	[13.8,15.6]		533 (16.9)	24.7	[23.2,26.2]
	55-64	1,339 (22.4)	20.3	[19.2,21.3]		778 (24.7)	16.6	[15.3,17.9]
	65 +	1,445 (24.1)	19.3	[18.3,20.3]		1,061 (33.7)	19.3	[17.9,20.7]

© 2019 Huang J et al. *JAMA Network Open.*

Race/Ethnicity								
	White, Non-Hispanic	4,365 (72.9)	64.3	[63.1,65.5]		1,868 (63.3)	65.7	[64.0,67.4]
	Black, Non-Hispanic	600 (10.0)	11.8	[11.0,12.6]		409 (13.9)	10.3	[9.2,11.4]
	Hispanic	639 (10.7)	15.8	[14.9,16.8]		427 (14.5)	15.8	[14.4,17.1]
	Other	388 (6.5)	8.0	[7.3,8.7]		249 (8.4)	8.3	[7.3,9.3]
Education								
	< High School	326 (5.4)	10.8	[10.0,11.6]		217 (6.8)	8.7	[7.7,9.6]
	High school graduate	1,345 (22.5)	28.9	[27.8,30.0]		616 (19.4)	22.9	[21.5,24.4]
	Some college	2,014 (33.6)	28.6	[27.5,29.8]		942 (29.6)	32.8	[31.2,34.5]
	College +	2,307 (38.5)	31.6	[30.5,32.8]		1,406 (44.2)	35.6	[33.9,37.2]
Household income								
	< 20K	789 (13.2)	11.8	[11.0,12.6]		559 (18.9)	17.4	[16.1,18.8]
	20 - 35K	807 (13.5)	12.2	[11.4,13.0]		423 (14.3)	12.2	[11.0,13.4]
	35 - 50K	731 (12.2)	11.0	[10.2,11.8]		386 (13.0)	14.9	[13.6,16.2]
	50 - 75K	1,076 (18.0)	17.4	[16.4,18.3]		530 (17.9)	19.1	[17.7,20.5]
	75K +	2,589 (43.2)	47.6	[46.4,48.9]		1,064 (35.9)	36.3	[34.6,38.1]
Marital status								
	Married	3,401 (56.8)	57.3	[56.1,58.6]		1,653 (52.2)	52.2	[50.5,54.0]
	Not married	2,591 (43.2)	42.7	[41.4,43.9]		1,513 (47.8)	47.8	[46.0,49.5]
Metro status								
	Non-metro	849 (14.2)	13.8	[12.9,14.7]		473 (14.4)	15.6	[14.4,16.9]
	Metro	5,142 (85.8)	86.2	[85.3,87.1]		2,812 (85.6)	84.4	[83.1,85.6]

© 2019 Huang J et al. *JAMA Network Open.*

*: E-cigarette use was not asked in HINTS in 2017.				
†: This option was asked only in the HINTS-FDA 2015 data. Other HINTS survey did not have this option.				

© 2019 Huang J et al. *JAMA Network Open.*
